# Psychosocial functioning in patients with treatment-resistant depression after group cognitive behavioral therapy

**DOI:** 10.1186/1471-244X-10-22

**Published:** 2010-03-16

**Authors:** Miki Matsunaga, Yasumasa Okamoto, Shin-ichi Suzuki, Akiko Kinoshita, Shinpei Yoshimura, Atsuo Yoshino, Yoshihiko Kunisato, Shigeto Yamawaki

**Affiliations:** 1 Department of Psychiatry and Neurosciences, Division of Frontier Medical Science, Programs for Biomedical Research, Graduate School of Biomedical Sciences, Hiroshima University, 1-2-3, Kasumi, Minami-ku, Hiroshima 734-8551, Japan; 2 Department of Social and Clinical Psychology, Faculty of Contemporary Culture, Hijiyama University, 4-1-1, Ushitashinmachi, Higashi-ku, Hiroshima, 732-8509, Japan; 3 Faculty of Human Sciences, Waseda University 2-579-15, Mikajima, Tokorozawa, Saitama 359-1192, Japan

## Abstract

**Background:**

Although patients with Treatment Resistant Depression (TRD) often have impaired social functioning, few studies have investigated the effectiveness of psychosocial treatment for these patients. We examined whether adding group cognitive behavioral therapy (group-CBT) to medication would improve both the depressive symptoms and the social functioning of patient with mild TRD, and whether any improvements would be maintained over one year.

**Methods:**

Forty-three patients with TRD were treated with 12 weekly sessions of group-CBT. Patients were assessed with the Global Assessment of Functioning scale (GAF), the 36-item Short-Form Health Survey (SF-36), the Hamilton Rating Scale for Depression (HRSD), the Dysfunctional Attitudes Scale (DAS), and the Automatic Thought Questionnaire-Revised (ATQ-R) at baseline, at the termination of treatment, and at the 12-month follow-up.

**Results:**

Thirty-eight patients completed treatment; five dropped out. For the patients who completed treatment, post-treatment scores on the GAF and SF-36 were significantly higher than baseline scores. Scores on the HRSD, DAS, and ATQ-R were significantly lower after the treatment. Thus patients improved on all measurements of psychosocial functioning and mood symptoms. Twenty patients participated in the 12-month follow-up. Their improvements for psychosocial functioning, depressive symptoms, and dysfunctional cognitions were sustained at 12 months following the completion of group-CBT.

**Conclusions:**

These findings suggest a positive effect that the addition of cognitive behavioural group therapy to medication on depressive symptoms and social functioning of mildly depressed patients, showing treatment resistance.

## Background

About 20 to 40% of depressed patients do not respond satisfactorily to treatment with only antidepressant medications [1-3]. These patients are defined as having treatment-resistant depression (TRD) when they fail to respond to at least two adequate trials of antidepressant medications from different classes [3,4].

TRD patients frequently have impaired social functioning because of sustained depressive symptoms [5]. The impairments affect marriages, cause interpersonal problems, and difficulty in work environments [6]. Continued depression and psychosocial impairment may induce social isolation, loneliness, and interpersonal difficulties that also interfere with the improvement of depressive symptoms [7]. TRD patients who received treatment as usual (TAU) with only medication continued to have functional disability [8].

Cognitive behavioral therapy (CBT) has been shown to be effective in the treatment of major depressive disorder. DeRubeis et al. [9] suggested that CBT can be as effective as medication for the initial treatment of moderate to severe major depression. Other studies have shown that adding CBT to medication for TRD may be beneficial in reducing depressive symptoms. For example, Thase et al. [10] compared the effectiveness of CBT and medication as second-step strategies for the patients with an unsatisfactory response to an initial trial of medication (citalopram). They reported that those patients who received CBT (either alone or in combination with citalopram) had similar response and remission rates to those who received only medication. However, these studies have mainly investigated the short-term effects of CBT on depressive symptoms. Several studies investigated whether CBT improved social functioning in individuals with chronic depression. Scott et al. [11] assessed psychological and social functioning, and compared medication management alone to CBT plus medication management. They reported that patients receiving cognitive therapy plus medication management had better psychosocial functioning than those who receiving medication management alone. Hirschfeld et al. [12] studied patients who underwent a cognitive behavioral analysis system of psychotherapy (CBASP) as CBT for chronic depression, and compared the efficacy of (1) CBASP, (2) nefazodone, or (3) CBASP combined with nefazodone for improving psychosocial functioning. They reported that the combined therapy had greater effects than either monotherapy. These studies have been limited to consideration of the short-term effectiveness of CBT for social functioning, and they did not necessarily meet criteria for treatment resistant.

Impaired social functioning may be a contributing cause as well as an effect of depression in individuals with TRD. Studies have not examined the effectiveness of CBT, along with medication, for patients with TRD with regard to both depressive symptoms and psychosocial functioning, particularly with longer-term follow-up. Therefore, we examined the short-term effectiveness of combined therapy (group-CBT and medication) on not only the depressive symptoms but the social functioning of mild TRD patients. Moreover, we studied these long-term effects (12 months) after the termination of group-CBT. We addressed the following questions:

1. Is the combined therapy (group-CBT and medication) effective in improving not only the depressive symptoms but the social functioning of patients with treatment-resistant depression?

2. Are these effects of the combined treatment for TRD maintained 12 months after termination of the group-CBT?

## Methods

### Participants

**Figure 1:**
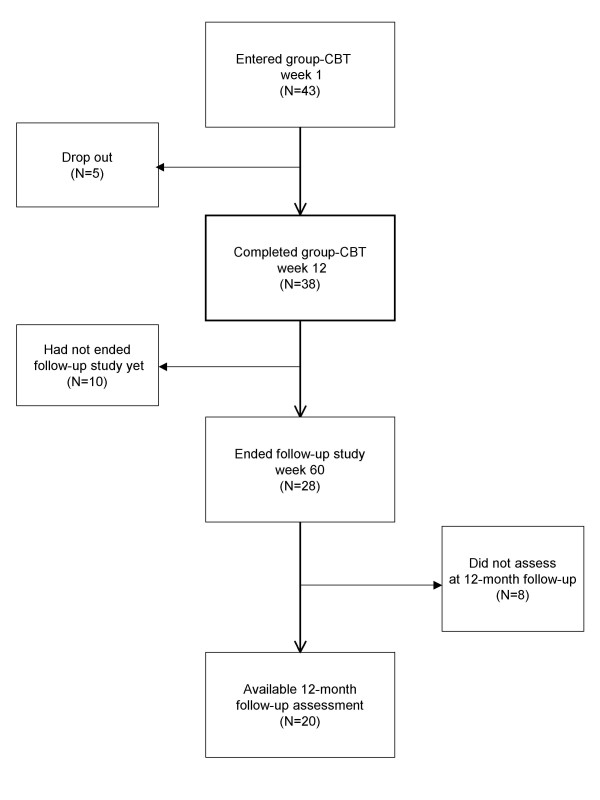
**Flow chart of participants**.

A flow chart of participants is shown in Fig. 1. Forty-three patients were recruited from the Department of Psychiatry and Neurosciences at Hiroshima University Hospital. Criteria for inclusion in the treatment study were: (a) outpatients who could participate in the group-CBT for 12 weeks, (b) a diagnosis of major depressive disorder for the current episode established by a psychiatrist or a clinical psychologist using the Structured Clinical Interview for DSM-IV(SCID) [13,14], (c) Hamilton Rating Scale for Depression (HRSD) [15] score of 8 or greater, and (d) patients being defined as the treatment resistant according to the staging system of antidepressant resistance [4], with the level of the treatment resistance at stage 2 or greater. Exclusion criteria were: current or previous diagnosis of a psychotic spectrum disorder, evidence of organic brain disorder, mental retardation, personality disorder, current high risk of suicide, substance abuse, or serious somatic disease. All patients were evaluated by a psychiatrist or a clinical psychologist using the Structured Clinical Interview for Axis I (SCID-I) [16] and the Structured Clinical Interview for Axis II (SCID-II) [17].

All patients had previously taken two different classes of antidepressant medications for a minimum of 8 weeks without remission of symptoms (some patients had mild depressive symptoms): clomipramine (n = 13, average 146 mg per day), paroxetine (n = 13, average 29 mg per day), milnacipran (n = 13, average 103 mg per day), or others. The drug type and dose was maintained during the group-CBT treatment. We defined patients whose medications were changed as dropouts. In addition, the patients did not take any other forms of treatment except medication for the 12 months after the group-CBT.

The study protocol was approved by the Ethics Committee of the Hiroshima University Graduate School of Medical Sciences (Reference number: 628). Written informed consent was obtained from all patients.

### Measures

The patients were assessed using the following instruments at pretreatment baseline, post-treatment, and 12 months after completion of the group-CBT.

#### a) Functioning assessment

The Global Assessment of Functioning (GAF: DSM-IV-TR) [13,14] and the 36-item Short-Form Health Survey (SF-36) [18,19] were used to measure social functioning and quality of life. The GAF provides a rating of psychological, social, and occupational functioning on a hypothetical continuum of mental health/illness rating from 0 to 100. A rating higher than 70 indicates no more than slight impairment in social, occupational or school functioning.

The SF-36 is a 36-item questionnaire about functional status and well being. The SF-36 is comprised of the Physical Component Summary (PCS) and the Mental Component Summary (MCS), each with 4 subscales: (1) physical functioning, (2) role-physical factor in functioning, (3) bodily pain, (4) general health, (5) vitality, (6) social functioning, (7) role-emotional factor in functioning, and (8) mental health. Each score ranges from 0 to 100, with 0 representing the poorest functioning and 100 representing optimal health. The Cronbach's alpha reliability estimates for the Japanese SF-36 are 0.71-0.87 for the subscales, indicating good test-retest reliability [20].

#### b) Depressive symptoms assessment

The Hamilton Rating Scale for Depression (HRSD) [15,21] is a 17-item scale used by the interviewing clinician to assess the patient's depressive symptoms. The test-retest reliability correlation is 0.81, which indicates adequate reliability [22].

#### c) Dysfunctional cognitions assessment

Dysfunctional cognitions were assessed by the Dysfunctional Attitude Scale (DAS) [23,24] and the Automatic Thought Questionnaire-Revised (ATQ-R) [25,26]. The DAS is a 40-item self-report inventory designed to measure unstated assumptions and maladaptive beliefs often found in depressed individuals. The Cronbach's alpha reliability estimate for the Japanese version is 0.86, consistent with good test-retest reliability [24].

The ATQ-R is a 40-item self-report scale designed to assess the levels of automatic thoughts. The ATQ-R comprises both negative and positive thought scales. The Japanese version has been tested in university students. The Cronbach's alpha reliability estimate of the Japanese version has been reported as 0.94 for the negative thought scale and 0.88 for the positive thought scale. Moreover, the sufficient reliability and construct validity of the scale has been reported [26].

### Treatment Procedures

Cognitive behavioral group therapy was conducted for 12 weekly 90-minute sessions; each group was comprised of five or six patients. The treatment was conducted by two psychotherapists (one was a doctoral level clinical psychologist with 12 years of experience, the other was a doctoral student psychologist with 4 years of experience) and a psychiatrist with 10 years of experience.

### Treatment Protocol

The treatment program was based on research conducted by Beck et al. [27]. The program consists of 12 structured sessions, as follows: (Session 1) Psycho-education about depression; (Session 2) Psycho-education about group-CBT; (Session 3) Instruction about self-monitoring thinking, behavior, and mood; (Session 4) Information about understanding the relationship between cognition and mood; (Session 5) Identifying the features of participants' own negative thinking; (Session 6) Challenging one's own negative thinking; (Session 7) Challenging and restructuring one's own negative thinking; (Session 8) Looking for new ideas and invoking positive thinking; (Session 9) Practicing the new ideas and positive thinking in daily life; (Session 10) Evaluating one's own ideas and thinking during the last week, and setting up an action plan for the next week; (Session 11) Reviewing the outcome of the program; (Session 12) A lecture on relapse prevention. The treatment program also used structured diaries and homework assignments.

### Statistical Methods

First we examined the differences between baseline and post-treatment using an analysis of covariance (ANCOVA) that controlled for the baseline levels of variables. We calculated the improvement effect sizes [partial η^2 ^= F * *df*_time_/F * *df*_time _+ *df*_error_] [28]. According to conventional criteria a partial η^2 ^of 0.01 is small, 0.06 is moderate, and 0.14 is large. Statistically significant differences were evaluated with paired *t*-tests (using a Bonferroni correction). If there were statistically significant differences, we also computed Cohen's *d *as a measure of the pre-post effect size. According to the criteria of Cohen's classification a *d *of 0.2 is small, 0.5 is medium, and 0.8 is large [29].

Next, we calculated the remission and response rates after the completion of the group-CBT. Remission was defined as a score of 7 or less on the HRSD. A positive treatment response was defined as a 50% or greater reduction in the HRSD score compared to the pre-treatment score. We also calculated the reliable change and clinically significant change of depressive symptoms using Jacobson and Truax's (JT) method, which uses two steps [30,31]. The first step is to define a cutoff point that separates the functional population from the dysfunctional population. The cutoff we used point was ± 2 SD from the pre-treatment mean. The second step compares an individual's change from pre- to post-treatment to a standard error of measurement of the outcome, referred to as the Reliable Change Index (RCI). If the RCI is higher than 1.96, the probability that the pre-post treatment difference occurred by chance is less than 5%. Using the results of these two steps, we classified patients into three categories: recovered (passed cutoff point and RCI >1.96), improved (did not pass cutoff point but RCI >1.96), or unchanged or deteriorated (passed neither criterion).

Finally, we analyzed the follow-up data using repeated measures ANCOVA for each outcome measurement (assessed at pre-treatment, post-treatment, and 12 months after group-CBT), and calculated improvement effect sizes. We performed repeated measures ANCOVA using pre-treatment scores as covariates. In the case of significant comparisons, we conducted post hoc paired *t*-tests using a Bonferroni correction.

All analyses were conducted on intent-to-treat (ITT) and completed treatment (Completer) samples. In the ITT analyses, the missing post-treatment or follow-up data were considered to be non-responders or adverse events, and their last available observations were carried forward (LOCF: last observation carried forward).

All statistical tests were two-tailed, with an alpha level of 0.05. All the data were examined using SPSS for Windows, version 16.0.

## Results

### Clinical backgrounds

Table 1 shows the demographic and clinical characteristics of the 43 patients enrolled in the group-CBT. There were 24 men and 19 women, mean age 41.3 years; 28 were married. The majority of patients had had more than 13 years of education (77%). The patient's average duration of depressive illness was 19.4 ± 15.6 months. Eighteen patients had not responded after treatment with two or more antidepressants with different action mechanisms (stage 2), and 25 patients (stage 3) had failed treatment with tricyclic antidepressants in addition to stage 2 criteria. Eighteen (42%) were experiencing their first depressive episode. The baseline scores on the Hamilton Rating Scale for Depression (HRSD) indicated mild to moderate levels of depression among the patients (Mean = 14.7, *SD *= 4.4). Seven patients (16%) had HRSD scores between 8 and 10, 13 (30%) had scores between 11 and 14, 13 (30%) had scores of between 15 and 18, and 10 (23%) had scores between 18 and 27. The baseline GAF scores indicated a poor level of social functioning. 31 patients (72%) had scores between 40 and 60, and the other 12 (28%) had scores between 61 and 70.

**Table 1:** Baseline demographic and clinical characteristic (N = 43)

	N	%
Female	19	44.2
Age at intake, mean(SD), year	41.3 (9.2)	
*Employ status*		
Employed	1	2.3
Absence from work	31	72.1
Unemployed	11	25.6
*Marital status*		
Single	15	34.9
Married	28	65.1
Education, mean (SD), year	14.9 (1.9)	
*Diagnosis*		
Single episode	18	41.9
Recurrent	25	58.1
*Treatment-resistant depression level*		
TRD level II	18	41.9
TRD level III	25	58.1
Number of episode, median (range)	2 (1-4)	
Duration of the current episode, mean (SD)	19.4 (15.6)	
HRSD score, mean (SD)	14.7 (4.4)	
GAF score, mean (SD)	59.5 (6.1)	

HRSD: Hamilton Rating Scale for Depression

GAF: Global Assessment of Functioning scale

Of the 43 patients who began the group-CBT, 38 completed the program and 5 dropped out. Four dropouts were due to worsening symptoms, and the fifth was dissatisfied with the program. The ITT sample is the total initial patient sample of 43, while the Completer sample is the 38 patients who completed the group-CBT. The demographic and clinical characteristics did not differ between those who completed the group-CBT and those who did not.

### Acute treatment outcomes

#### a) Functional status

Table 2 displays the results of ANCOVAs for the GAF and Short-Form Health Survey (SF-36) scores from pre- to post-treatment. For both the ITT and Completer analyses, the GAF scores increased significantly (ITT: *F *(1, 83) = 40.06, *p *< 0.001, partial η^2 ^= 0.33, Cohen's *d *= 0.94; Completer: *F *(1, 72) = 41.19, *p *< 0.001, partial η^2 ^= 0.53, Cohen's *d *= 1.24). For the ITT sample, the number of patients who were rated as showing mild functional impairment (defined as GAF scores over 60) improved from 12 (28%) at baseline to 30 (70%) at post-treatment; 7 (16%) of these patients were rated as having minimal impairment (GAF > 70). As expected, the Completer sample comprised those 30 patients who had a post-treatment GAF score over 60 (79%), and the 7 patients (18%) who were rated as having minimal impairment (GAF > 70).

**Table 2:** ANCOVAs of treatment outcome as measured by GAF and SF-36

	pre *Mean(SD)*	post treatment *Mean(SD)*	*F value*	effect size *partial Eta*^2^	*d*
*ITT(N = 43)*					
GAF	59.49(6.10)	65.51(6.68)	40.06***	0.33	0.94
SF-36 PCS	41.00(11.12)	46.78(10.22)	16.31***	0.16	0.54
Physical functioning	76.33(17.38)	84.77(15.92)	16.49***	0.17	0.51
Role physical	27.33(42.19)	58.14(42.86)	5.14*	0.06	0.72
Bodily pain	61.58(25.68)	68.45(26.94)	4.08*	0.05	0.26
General health	38.66(15.76)	48.74(20.36)	5.73*	0.07	0.00
SF-36 MCS	25.77 (8.70)	33.52(10.97)	25.17***	0.23	0.78
Vitality	24.65(13.69)	39.65(19.32)	11.58**	0.12	0.90
Social functioning	43.84(20.79)	59.59(23.75)	16.15***	0.16	0.71
Role emotional	11.63(28.06)	34.88(39.14)	19.06***	0.19	0.68
Mental health	37.86(15.82)	52.09(18.34)	30.24***	0.27	0.83

*Completer (N = 38)*					
GAF	60.18(5.79)	67.00(5.17)	41.19***	0.53	1.24
SF-36 PCS	41.18(11.04)	47.72(9.62)	17.73***	0.20	0.63
Physical functioning	77.43(16.65)	86.97(13.78)	26.38***	0.27	0.62
Role physical	23.68(41.08)	58.55(43.21)	47.11***	0.40	0.83
Bodily pain	62.68(26.63)	70.46(27.55)	4.15*	0.05	0.29
General health	38.62(16.03)	50.03(20.76)	13.23**	0.15	0.62
SF-36 MCS	25.93 (8.95)	34.71(10.90)	27.53***	0.27	0.88
Vitality	24.87(12.81)	41.84(18.25)	34.89***	0.32	1.08
Social functioning	44.34(21.83)	62.17(23.87)	17.21***	0.19	0.78
Role emotional	11.40(29.28)	37.72(40.40)	20.19***	0.22	0.75
Mental health	38.00(16.10)	54.11(17.93)	33.73***	0.32	0.95

** *p *< .001, ** *p *< .01, * *p *< .05

GAF: Global Assessment of Functioning scale

SF-36: 36-item Short-Form Health Survey

PCS: Physical Component Summary

MCS: Mental Component Summary

On the SF-36, the physical health (PCS) and mental health (MCS) scores at post-treatment were higher than at baseline, for both the ITT and Completer samples (all *p *values < 0.01). The effect sizes of the MCS improvement were greater than these for the PCS, indicating that the group-CBT was more strongly associated with improvement in mental health than physical health. Seven of the 8 subscale scores were improved significantly (bodily pain was not). The pre-post effect sizes (Cohen's *d*) for the vitality (ITT: 0.90; Completer: 1.08) and mental health (ITT: 0.83; Completer: 0.95) subscales were especially larger than for the other subscales.

#### b) Depressive symptoms

ANCOVA for the Hamilton Rating Scale for Depression (HRSD) scores showed a highly significant time effect. For the ITT sample, the mean HRSD scores decreased from 14.7 at pre-treatment to 9.2 at post-treatment (*F *(1, 83) = 42.23, *p *< 0.001, partial η^2 ^= 0.34, Cohen's *d *= 1.09). For the Completer, the mean HRSD scores decreased from 14.2 at pre-treatment to 8.2 at post-treatment (*F *(1, 73) = 53.29, *p *< 0.001, partial η^2 ^= 0.42, Cohen's *d *= 1.30). Among the Completers, 21 (55%) of the patients had scores of 7 or less on the HRSD at post-treatment. 12 had scores between 8 and 14, and 5 had scores between 15 and 21.

Table 3 shows the remission and response rates at post-treatment for the ITT and Completer sample analyses. Twenty-one participants (ITT: 49%; Completer: 55%) met criteria for remission (HRSD score of 7 or less), and 18 participants (ITT: 42%; Completer: 47%) showed at least a 50% reduction of their scores on the HRSD from the pre-treatment score. The number of participants who met criteria both for remission and 50% reduction of were 17 (ITT: 40%; Completer: 45%).

**Table 3:** Remission and response rates after group-CBT

Outcome	Criteria	N		%
Remission				
	HRSD score ≦7 ITT (N = 43)	21		48.8
	HRSD score ≦7 Completers (N = 38)	21		55.3
Response				
	HRSD score reduction of ≧50% ITT (N = 43)	18		41.9
	HRSD score reduction of ≧50% Completers (N = 38)	18		47.4

In addition, we calculated the reliable change and clinically significant change using Jacobson and Truax's formula [30]. For the ITT sample, the cutoff point on the HRSD was 5. The criteria for "recovered" were fulfilled by 9 (21%) participants, 10 (23%) were "improved", and 24 (56%) were "unchanged" or "deteriorated". Among the 38 patients who completed the treatment, the cutoff point on the HRSD was 6. Nineteen (50%) met criteria for "recovered" or "improved", and the other 19 (50%) were classified as "unchanged or deteriorated".

#### c) Dysfunctional cognitions

The score on the Dysfunctional Attitude Scale (DAS) decreased significantly from pre-treatment to post-treatment for both the ITT and Completer samples. The mean of the DAS scores changed using the LOCF (last observation carried forward) method from 161.3 to 147.6 (*F *(1, 83) = 17.13, *p *< 0.001, partial η^2 ^= 0.29, Cohen's *d *= 0.17). The mean for the 38 in the Completer sample decreased from 156.3 to 140.9 (*F *(1, 73) = 18.42, *p *< 0.001, partial η^2 ^= 0.20, Cohen's *d *= 0.48).

In addition, the means on the ATQ-R negative scale at post-treatment were significantly lower than the means at pre-treatment using the same two methods of analysis. The mean of the ATQ-R negative scale scores changed using the LOCF method from 90.0 to 70.8 (*F *(1, 83) = 39.09, *p *< 0.001, partial η^2 ^= 0.32, Cohen's *d *= 0.76). The mean for the 38 in the Completer sample decreased from 87.2 to 65.5 (*F *(1, 73) = 50.61, *p *< 0.001, partial η^2 ^= 0.41, Cohen'd = 1.00). However, there was no significant difference on the ATQ-R positive scale between pre-treatment and post-treatment in the ITT or Completer sample analyses.

### A 12-month follow-up outcome

Of the 38 patients who completed the group-CBT, a total of 28 patients had completed the treatment more than one year previously at the time of our follow-up. The remaining 10 persons had completed the group-CBT were less than one year previously at the time of our follow-up. Twenty of the 28 patients (71%) completed all measurements one year after finishing the group-CBT; the other 8 refused to participate in the follow-up (4 refused the participation in the follow-up study, and 4 refused accesses to contact for follow-up).

We analyzed the follow-up data using both the ITT and Completer samples. For the ITT analysis which included 12 dropouts (4 who did not complete the treatment, and 8 who refused the follow-up study), the last observation values were carried forward (LOCF). We excluded one patient who dropped out from the ITT samples because the patient had not passed for one year at the time of the follow-up assessment. Table 4 shows the changes in functional status measured by the GAF and SF-36 for the ITT and Completer samples. The repeated measures ANCOVAs for GAF revealed a significant time effect for the both the ITT sample and Completer samples (both p value < 0.001). Post hoc paired *t*-tests with a Bonferroni correction showed that the score at post-treatment was higher than the score at baseline, and the score at the 12-month follow-up was also higher than at the post-treatment (*p *< 0.001). For the ITT sample, including the 12 dropouts, 27(84%) met criteria for the mild-minimal impairment (GAF < 60), and 12 patients (38%) reached the level of functioning well. For the Completer sample, except for one patient, all patients (95%) were at the mild-minimal impairment level (GAF < 60), and 10 patients (50%) were functioning well (GAF > 70) at the 12-month follow-up.

**Table 4:** Repeated measure ANCOVAs of 12-month follow-up measured by GAF and SF-36

	Pre-treatment *Mean (SD)*	Post-treatment *Mean (SD)*	12 months *Mean (SD)*	*F value*	effect size *partial Eta*^2^
*ITT (N = 32)*					
GAF	60.62(6.36)	66.41(6.74)^a^	71.22(9.16)^b, c^	21.44***	0.32
SF-36 PCS	42.23 (8.88)	48.21 (9.78)^a^	46.97 (9.34)^b^	6.71**	0.13
Physical functioning	76.87(16.84)	84.53 (14.67)^a^	84.53 (14.11)^b^	8.28***	0.15
Role physical	21.88(39.53)	60.16 (45.73)^a^	59.38 (40.54)^b^	11.39***	0.20
Bodily pain	65.84(22.56)	70.73 (25.29)	74.06 (24.06)	1.87	0.04
General health	39.78(14.92)	51.13 (20.59)^a^	49.91 (19.64)^b^	5.19**	0.10
SF-36 MCS	26.16 (9.70)	33.59 (11.20)^a^	38.34 (11.63)^b^	15.32***	0.25
Vitality	26.41(14.66)	40.31 (19.10)^a^	43.75 (19.76)^b^	12.59***	0.22
Social functioning	44.84(21.91)	60.55 (23.14)^a^	68.75 (25.79)^b^	10.51***	0.19
Role emotional	13.54(31.52)	37.50 (42.12)^a^	54.17 (43.79)^b^	13.07***	0.22
Mental health	38.13(16.51)	51.63 (18.16)^a^	55.83 (18.80)^b^	13.18***	0.22

*Completers (N = 20)*					
GAF	60.24 (6.86)	66.48 (6.40)^a^	73.81 (7.29)^b, c^	25.99***	0.48
SF-36 PCS	43.80 (8.48)	50.32 (7.88)^a^	48.18 (7.28)	5.10**	0.15
Physical functioning	80.25(13.13)	89.50 (9.02)^a^	89.00 (7.88)^b^	12.31***	0.31
Role physical	21.25(40.78)	57.50 (47.37)^a^	56.25 (38.79)^b^	5.75**	0.17
Bodily pain	66.60 (24.30)	73.28 (27.40)	76.30 (26.75)	1.34	0.05
General health	39.55 (16.17)	55.90 (22.06)^a^	54.10 (21.19)^b^	6.94**	0.27
SF-36 MCS	25.23 (9.77)	33.78 (11.06)^a^	40.56 (10.71)^b, c^	13.91***	0.33
Vitality	25.50 (13.66)	42.25 (16.58)^a^	47.25 (17.66)^b^	10.78***	0.28
Social functioning	48.00 (21.86)	61.87 (26.12)	73.13 (29.32)^b^	8.69**	0.24
Role emotional	13.33 (33.16)	40.00 (45.37)	63.33 (45.76)^b, c^	11.23***	0.29
Mental health	34.80 (16.68)	52.00 (16.57)^a^	57.60 (17.38)^b^	15.00**	0.35

*** *p *< .001, ** *p *< .01, * *p *< .05

a: significant difference between Pre- and Post-treatment (*p *< .05)

b: significant difference between Pre- and 12 months after treatment (*p *< .05)

c: significant difference between Post- and 12 months after treatment (*p *< .05)

The repeated measures ANCOVAs for the SF-36 (both PCS and MCS) also showed significant time effects for both the ITT samples and the Completer samples (all *p *values < 0.01). Post hoc paired *t*-tests with a Bonferroni correction demonstrated that MCS scores at post-treatment and at 12-month follow-up were higher than the baseline score in the Completer analysis (*p *< 0.001). However, in the ITT analysis, MCS score at follow-up was not significantly difference from that at post-treatment. Regarding the subscale scores, 7 of the 8 subscale scores at the 12-month follow-up were significant higher than the baseline scores (bodily pain was the exception).

Regarding depressive symptoms, in both the ITT and Completer analyses, there was a significant change in the Hamilton Rating Scale for Depression (HRSD) score during the 12-month follow-up (both *p *values < 0.001), with the score at the follow-up being lower than the score at baseline (*ps *< 0.001). For the ITT sample, 22 patients (69%) had scores of 7 or less on the HRSD at the 12-month follow-up. 8(25%) had scores between 8 and 14, and other two had scores between 15 and 22. Of the 20 in the Completer sample, 14 (70%) had scores of 7 or less on the HRSD at the 12-month follow-up. Five (25%) had scores between 8 and 14, and one scored 22.

Additionally, dysfunctional cognitions measured by the DAS and the ATQ-R negative scales showed sustained improvements in both the ITT and Completer analyses (all *p *values < 0.05). However, there was no significant change on the ATQ-R positive scale in the ITT or Completer sample analyses.

## Discussion

We examined the efficacy of the adding cognitive behavioral therapy to treatment with medication for improving both the depressive symptoms and the social functioning of TRD patients. The baseline scores on the HRSD in the present study were in the mild to moderate depression range. However, psychosocial functioning in the majority of patients was poor. The mean of the enrolled patients' depressive episode at the baseline was 19.4 months, which indicated that their depressive symptoms and psychosocial functioning impairments had existed for long term. The cognitive behavioral therapy combined with medication for the patients with TRD resulted in significant improvement in both the depressive symptoms and the social functioning of the patients, and maintained improvement after a one year follow-up. As far as we know, this is the first study to investigate the long term effectiveness of adding cognitive behavioral therapy to medication for improving both depressive symptoms and social functioning of patients defined as TRD.

Few previous studies have investigated the social functioning of patients with TRD. Dunner et al. [8] assessed the social functioning of TRD patients (N = 124), using the SF-36 with treatment as usual (TAU) over two years. They reported that the scores on the PCS and MCS scales of the SF-36 did not change over the two years. In the present study, the PCS and MCS scores were similar at baseline to the results of Dunner et al. [8], but these scores in our study showed sustained improvement, especially for the mental components (MCS), after CBT treatment and one year later. For example, the MCS score at 12 months in the Dunner et al. [8] study was 27.8, while in our study it was 40.6. In combination with the findings of Dunner et al., these findings indicate a possibility that combining cognitive behavioral group therapy with medication improves social functioning more than TAU. In our study, the vitality subscale and the mental health subscale scores were especially increased. The improvements support the hypothesis that CBT may be promoting an improvement in energy or vitality via an increase in overall activity level [32].

In recent years, social functioning has become of increasing importance in the treatment and outcome assessment of psychiatric disorders. Some researchers have suggested that a broader definition of remission is needed - one that involves not only the absence of symptoms but also improvement in psychosocial functioning [33,34]. They emphasize that the improvement in psychosocial functioning may be necessary not only to prevent relapse but also to ensure full remission of the disorder. At the same time, interest in CBT approaches as an effective intervention to improve psychosocial recovery is also increasing. There are trials of CBT focused on social functioning in individuals with bipolar disorder [32], bulimia nervosa [35], and psychosis [36]. In the case of chronic depression, several studies reported the short-term effectiveness of CBT in improving social functioning [11,12]. It is likely that chronic depression in these studies included treatment-resistant depression. Our study indicates that the improvements in social functioning are sustained over one year after CBT.

Our protocol used basic CBT strategies, and did not include social skills training or stress management. However, the patients learned appropriate cognitive and behavioral coping strategies for increasing meaningful activity and managing interpersonal stress [32,37]. The group-CBT provided both social support and also modeling effect [38,39]. The group format provided patients with opportunities for practicing new cognitive and behavioral skills, which they could apply in their lives after completion of the group-CBT [39]. These cognitive and behavioral skills may have influenced the improvement of social functioning.

Regarding depressive symptoms, about 50 to 55% of the participants who completed the group-CBT sessions achieved remission after the completion of treatment (HRSD score of 7 or less), and about 40 to 50% of the participants were judged to be responders (HRSD score decreased by 50%). In terms of the clinical significant change [30], half of the patients showed recovery or improvement. These results are similar to the outcomes in previous studies (e.g. Fava et al.; Moore & Blackburn; Thase et al.) [10,40,41].

This study has several limitations. First, the lack of a control group limits the interpretation of the results. It remains unknown whether the improvement in social functioning with TRD is related to natural course of depression. In addition, it is not clear whether the group affiliation or the CBT strategy is the active factor accounting for the improvements. More research using a TAU (treatment as usual) control group or different treatment groups is needed. Second, most TRD patients in the present study were less severely depressed than in previous studies of patients with TRD [11,12,40], although they met diagnostic criteria for mild to moderate depression. So we do not know whether the findings of this study can be generalized to patients with severe TRD. Third, there were missing data from people who did not complete treatment. We used not only the completer analyses but also the ITT analyses. Although the results did not differ much between the dropouts (included in the ITT sample) and the treatment completers, there were some patients who did not complete group-CBT because they got worse. Also, control of the specific antidepressants could not be implemented in our longitudinal study. Therefore, the results of maintaining improvement may include some effects of medication.

Despite these limitations, the present study suggests that using group-CBT along with medication has a positive effect on both depressive symptoms and psychosocial functioning, a suggestion that needs to be confirmed in larger samples using randomized controlled trials.

## Conclusions

This study suggests a positive effect that combining cognitive behavioral therapy with medications improves both depressive symptoms and social functioning with TRD. Moreover, these improvements in both depressive symptoms and social functioning were maintained over one year following completion of CBT while continuing on medication.

## Abbreviations

ATQ-R: (Automatic Thought Questionnaire-Revised); CBT: (cognitive behavioral therapy); DAS: (Dysfunctional Attitude Scale); GAF: (Global Assessment of Functioning); HRSD: (Hamilton Rating Scale for Depression); ITT: (intent-to-treat); LOCF: (last observation carried forward); MCS: (Mental Component Summary); PCS: (Physical Component Summary); RCI: (Reliable Change Index); SF-36: (the 36-item Short-Form Health Survey); TRD: (treatment-resistant depression); TAU: (treatment as usual).

## Competing interests

The authors declare that they have no competing interests.

## Authors' contributions

MM participated sufficiently in the work to take responsibility for the entire content. YO and SYamawaki contributed to obtaining funding and critical revision of the manuscript. Authors SS, AK, SYoshimura, YK, and AY contributed to the clinical investigation (diagnosis, treatment and assessments). Authors YO, SS and SYamawaki contributed to the conceptualization and design of the study. All authors contributed to and have approved the final manuscript.

## Pre-publication history

The pre-publication history for this paper can be accessed here:

http://www.biomedcentral.com/1471-244X/10/22/prepub
